# Molecular iodine exerts antineoplastic effects by diminishing proliferation and invasive potential and activating the immune response in mammary cancer xenografts

**DOI:** 10.1186/s12885-019-5437-3

**Published:** 2019-03-22

**Authors:** Irasema Mendieta, Rosa E. Nuñez-Anita, Mario Nava-Villalba, Xóchitl Zambrano-Estrada, Evangelina Delgado-González, Brenda Anguiano, Carmen Aceves

**Affiliations:** 1 Instituto de Neurobiología, UNAM-Juriquilla, 76230 Querétaro, Mexico; 2 Facultad de Medicina Veterinaria y Zootecnia UMSNH, Michoacán, Mexico

**Keywords:** Molecular iodine, Mammary cancer, MCF-7, MDA-MB231, MCF-12F, Immune response, PPARγ, Iodine, Xenografts

## Abstract

**Background:**

The immune system is a crucial component in cancer progression or regression. Molecular iodine (I_2_) exerts significant antineoplastic effects, acting as a differentiation inductor and immune modulator, but its effects in antitumor immune response are not elucidated.

**Methods:**

The present work analyzed the effect of I_2_ in human breast cancer cell lines with low (MCF-7) and high (MDA-MB231) metastatic potential under both in vitro (cell proliferation and invasion assay) and in vivo (xenografts of athymic nude mice) conditions*.*

**Results:**

In vitro analysis showed that the 200 μM I_2_ supplement decreases the proliferation rate in both cell lines and diminishes the epithelial-mesenchymal transition (EMT) profile and the invasive capacity in MDA-MB231. In immunosuppressed mice, the I_2_ supplement impairs implantation (incidence), tumoral growth, and proliferation of both types of cells. Xenografts of the animals treated with I_2_ decrease the expression of invasion markers like CD44, vimentin, urokinase plasminogen activator and its receptor, and vascular endothelial growth factor; and increase peroxisome proliferator-activated receptor gamma. Moreover, in mice with xenografts, the I_2_ supplement increases the circulating level of leukocytes and the number of intratumoral infiltrating lymphocytes, some of them activated as CD8+, suggesting the activation of antitumor immune responses.

**Conclusions:**

I_2_ decreases the invasive potential of a triple negative basal cancer cell line, and under in vivo conditions the oral supplement of this halogen activates the antitumor immune response, preventing progression of xenografts from laminal and basal mammary cancer cells. These effects allow us to propose iodine supplementation as a possible adjuvant in breast cancer therapy.

**Electronic supplementary material:**

The online version of this article (10.1186/s12885-019-5437-3) contains supplementary material, which is available to authorized users.

## Background

Metastasis is a major cause of death in patients diagnosed with breast cancer [1]. Epithelial-mesenchymal transition (EMT) is a complex process that confers tumorous cells the ability to detach from the original tumor site. This process is accompanied by a reduced expression of proteins involved in cell-cell adhesion, including E-cadherin, and an increase in proteins associated with cell motility and invasion, such as vimentin and CD44 [2]. Invasiveness requires the activation of components like matrix metalloproteinases and mediators of angiogenesis and metastasis, including vascular endothelial growth factor (VEGF) and transforming growth factor β (TGF-β) [3–5]. Other signals recently recognized as essential for cancer promotion and metastasis are the pro-inflammatory factors present in the tumor microenvironment [6]. Several studies have shown that the immune system participates differentially in either elimination or progression of tumor cells, yet the equilibrium between these two effects has not been well established [7]. Some authors suggest that neoplastic cells and B cells suppress the immune system, promoting the proliferation and differentiation of lymphocytes to the regulatory T (Treg, FOXP3+) phenotype and the up-regulation of macrophage scavenger receptor 1 (MSR1) of dendritic cells, thus maintaining the inflammatory environment and promoting T cell exhaustion in T effector cells [8–10]. These conditions facilitate intravasation into the circulatory system and allow metastatic dissemination of the cancer cells [6, 10]. On the other hand, recent data have shown that restoring T cell function is possible with therapies that include radiation, vaccination (Her2 or immune reactive products), or metronomic chemotherapy [10–13]. The mechanisms by which these therapies enhance the proliferation, differentiation, and activity of the anti-tumor immune response could involve modifications in both tumoral and immune cells. For instance, there are reports on the induction of anti-angiogenic effects, re-differentiation or cellular death, and/or activation of cellular stress pathways that could reinitiate a targeted immune response at tumoral levels [12]. Studies concerning the immune system have demonstrated the direct reduction of Treg cells by inhibiting their suppressive function; as well as the induction of proliferation/differentiation/maturation of lymphocyte helper type 1 (Th1, CD4+), cytotoxic (CTL, CD8+), natural killer (NK, CD56+), and/or dendritic cells in antitumor immune re-activation [13].

Previous studies have shown that molecular iodine (I_2_) exerts an antineoplastic effect, triggering apoptotic and re-differentiation mechanisms in several cancer cells [14]. In addition, I_2_ has been proposed as an immune modulator. It is demonstrated that several immune cell types can internalize iodine, which could act as an antibacterial, anti-inflammatory, or as an inductor of immune response depending on the cellular context [15, 16]. However, specific effects of this element on tumor immune response have not been evaluated. The goal of this study was to analyze the role of I_2_ in the proliferation and invasive potential of human breast cancer cell lines with low (MCF-7) and high (MDA-MB231) metastatic potential under both in vitro (cell proliferation and invasion assay) and in vivo (xenografts) conditions. Athymic nude Foxn1 nu/nu mice were used as a model of reactivation of the immune response; despite the congenital absence of a thymus in these animals, the few T cells present in the spleen and lymph nodes are susceptible to stimulation and rouse an antitumor response [17, 18].

## Methods

### Materials

Dulbecco’s Modified Eagle’s Medium (DMEM), fetal bovine serum (FBS), penicillin, streptomycin, and trypsin-EDTA solutions were provided by GIBCO-BRL (Grand Island, NY, USA). Sublimed iodine was obtained from J.T. Baker (Estado de México, México). The concentration of iodine solutions was confirmed by titration with sodium thiosulfate [19].

### Cell culture and I_2_ effect in vitro

Normal MCF-12F (CRL-10783), tumorous estrogen receptor-positive MCF-7 (HTB-22), and triple-negative MDA-MB-231 (HTB-26) human breast epithelial cells were purchased from American Type Culture Collection (ATCC, Manassas, VA, USA). All cells were maintained in DMEM supplemented with 10% FBS, 100 U/ml penicillin, and 100 μg/ml streptomycin (basal medium) at 5% CO_2_ and 37 °C for 24 h before treatments.

### MTT viability assay

Cell viability was determined by 3-(4, 5-dimethylthiazol-2-yl)-2, 5-diphenyltetrazolium bromide (MTT) assay. Cells were seeded at 5 × 10^3^ cells/well in DMEM basal medium and incubated at 37 °C in 96-well plates with 5% CO_2_. After incubation overnight, the basal medium in each well was replaced with media containing one of the several concentrations of I_2_ and incubated for 48 h; 20 μL of MTT (5 mg/mL in PBS) was added to each well, and cells were incubated for 4 h at 37 °C. The supernatants were removed, 200 μL of dimethyl sulfoxide (DMSO) was added, and the absorbance (570 nm) was determined by a microplate reader (Bio-Rad, Hercules, CA, USA). Cell viability was expressed as percent change over control and calculated using the formula: (mean OD of treated cells/mean OD of untreated control cells) × 100.

### Invasive capacity

Invasive capacity was measured using a Transwell chamber coated with 30 μL Matrigel (reconstituted basement membrane; BD Biosciences, Mississauga, Canada). MDA-MB231 cells (2.5 × 10^4^ per Transwell) were incubated in a basal medium made with 200 μM I_2_ or deionized water, in 5% CO_2_ and at 37 °C. Cells in the upper chamber were removed with a cotton swab after 24 h. The cells remaining in the membrane were fixed using methanol for 10 min, then stained with 1% crystal violet solution and washed with PBS. The number of invading cells per field of view was counted at 20X magnification.

### Xenografts and I_2_ effect in vivo

Sixty-four female athymic hetero- and homozygous nude mice (Foxn1 nu/nu; Harlan Mexico, CDMX, Mexico) were kept in the specialized vivarium facility (semi-barrier) under controlled temperature conditions (22 ± 1 °C) on a 12-h:12-h light-dark cycle at 50% humidity. The home cage (Super Mouse AllerZone™, Micro-Isolator™ 1800™, LabProducts Inc. Houston, TX, USA) contained air filters, and mice were allowed ad libitum access to food (Purina certified rodent chow, Ralston Purina Co., St. Louis, MO, USA) and water. The procedures followed the Animal Care and Use Program of the National Institutes of Health (NIH, Bethesda, MD, USA) and were approved by the Research Ethics Committee of the Institute of Neurobiology (INB-UNAM) (Protocol #035).

All procedures were performed in the morning (8:00 am to 12:00 pm). Cells were implanted, and mice were sacrificed under anesthesia with a mixture of ketamine/xylazine (30 and 6 mg/kg body weight, respectively)*.* When homozygous nude mice were six weeks old, they were injected subcutaneously with 5 × 10^6^ cells of either the MCF-7 or the MDA-MB231 cell line in 50 μl PBS and 50 μl Matrigel. At 48 h post-inoculation, the mice were randomly divided into the control and iodine-treated groups (10–12 animals per group) (Additional file 1). Drinking water for the control group was deionized (6.76 ± 1.0 ml/animal/day) and supplemented with 0.025% I_2_ (6.4 ± 1.2 ml/animal/day) for each cage (3 animals per cage) of the iodine group (1.35–1.62 mg I_2_/animal/day). All animals were monitored every week for six weeks to identify body weight gain and tumoral growth. Tumor sizes were calculated by the ellipsoid formula [19]. In all animals, a necropsy was performed after sacrifice in open total body and in three tissues (e.g., lung, liver or skin around the tumors) to screen for possible metastases or alterations (e.g., ascites, inflammation, constipation).

To analyze the effect of iodine on the general immune response, hetero- and homozygous 6-week-old mice (10 animals per group) were supplemented with deionized water or 0.025% I_2_ for 6 weeks. Mice were sacrificed by decapitation, and blood was collected separately in sterile tubes with EDTA. White blood cells were counted using a hemocytometer. Ten microliters of blood were diluted 1:20 in Türk’s solution (Hycel Lab, Edo Mexico, Mexico) based on leukocyte staining with gentian violet.

### Immunohistochemistry for proliferating cell nuclear antigen and immunofluorescence for CD8+

First, three-micron slices of xenografts were deparaffinized and treated with 10 mM sodium citrate for 20 min at 80 °C for antigen retrieval. After maintaining at 25 degrees for cooling, slices were incubated with 0.3% hydrogen peroxide for blocking the activity of endogenous peroxidase. Non-specific binding was prevented with 2% non-fat dried milk in PBS buffer with 20% fetal bovine serum (at 37 °C for 1 h). Slices were incubated with the anti-rat monoclonal antibody against proliferating cell nuclear antigen (PCNA) generated in mouse, clone PC10 (DakoCytomation, Carpinteria, CA, USA). Followed of an incubation in a humid chamber at room temperature for 30 min. Immunocomplexes were identified by using goat anti-mouse antibody, coupled to peroxidase (EnVision™ + System, peroxidase, DakoCytomation, Carpinteria, CA, USA). The complex of Diaminobenzidine (DAB)/peroxidase/hematoxylin was used as the chromogenic substrate and was mounted (Merk, Darmstadt, Germany). As isotype control antibodies, tumor slices were incubated without either the primary or secondary antibody. The percentage of PCNA-positive cells was quantified by counting the number of labeled cells in at least 500 cells per region, and three random regions were analyzed [19].

CD8+ lymphocytes were analyzed in MDA-MB231 xenografts, and a lymph node from heterozygotic mice was used as a positive control. Slide preparation was carried out for visualization by confocal microscopy (Zeiss LSM 780; Jena, Germany). After antigen retrieval, slides were incubated for 20 min in 70% ethanol with 0.1% Sudan Black B (Sigma-Aldrich, Estado de Mexico, Mexico) to reduce autofluorescence. Detection of CD8 protein was carried out by primary monoclonal antibody (monoclonal CD8-PE 55032, 1:50, BD Biosciences, USA), and nuclei were observed using DAPI (1:100, Life Technologies). Slides were mounted using 4% antifade reagent (4% n-propyl gallate, 1% DABCO) and observed at 63X magnifications. Samples were excited using 633 nm (1.0% laser) and 488 nm (1.0% laser) wavelengths. Quantification was done by counting the CD8+ lymphocytes per field (Z-stack projection) and reported as the mean of the three fields randomly taken for each tumor.

### Histopathological analysis

Fixed xenografts were embedded in paraffin. From each block, sections of 3-μm width were cut and then placed on slides treated with 3-aminopropyltriethoxysilane. Hematoxylin and eosin (H&E) staining was used to observe histopathological change, especially lymphocytic infiltration and necrotic areas. The number of lymphocytes was evaluated by two independent observers (XZ and CA) in anonymized and blinded samples. The average of three random regions at 40X magnification was analyzed and the quantification was performed using ImageJ software (version 1.41, National Institutes of Health, Bethesda, MD, USA).

### Quantitative real-time PCR (RT-qPCR)

Gene expression was quantified by the qPCR method previously described [19]. Total RNA was obtained according to protocol described by the manufacturer (TRIzol reagent, Life Technologies, Inc., Carlsbad, CA, USA). 2 μg total RNA were reverse transcribed (RT) using oligo-deoxythymidine primers. To eliminate the genomic DNA, the RT assay was carried out for each sample, as a control one tube with a pool of all samples without transcriptase enzyme (−RT) was used. Each PCR was made using a specific pair of oligos detailed in Table 1. The Rotor-Gene 3000 apparatus (Corbett Research, Mortlake, NSW, Australia) was employed to perform qPCR with a marker for DNA amplification (SYBR Green, Fermentas, Burlington, ON, Canada). Relative expression of genes was calculated by using a standard curve and normalized to β-actin expression, as a housekeeping gene. In all RT-qPCR assays, the coefficient of variation for each gene was less than 16%, demonstrating that changes observed in different experimental groups correspond to changes in the genes.

**Table 1:** Oligonucleotides

Gen	Reference			bp
CD44	XM_017018585.2	AGAAGGTGGGCAGAAGAA	AAATGCGACCATTTCCTGAGA	115
CD24	XM_024446293.1	CCCACGCAGATTTATTCCAG	GACTTCCAGACGCCATTTG	255
E-cad	NM_001317186.1	TGCCCAGAAAATGAAAAAGG	GTGTATGTGGCAATGCGTTC	200
Vim	NM_003380.4	GAGAACTTTGCCGTTGAAGC	GCTTCCTGTAGGTGGCAATC	163
uPA	XM_011539867	GTGGCCAAAAGACTCTGAGG	CAAGCGTGTCAGCGCTGTAG	267
uPAR	XM_011527031	CAGACTTGCTGTGTGACCTCA	AATAACAACAACACAACAGCGG	183
VEGF	NM_001204384	GGCCTCCGAAACCATGAACTT	CCTCCTGCCCGGCTCACCCGC	165
TGF-β	NM_000660	CCCAGCATCTGCAAAGCTC	GTCAATGTACAGCTGCCGCA	100
PPARγ	XM_011533844	TCTCTCCGTAATGGAAGACC	CTTCACAAGCATGAACTCCA	325
β-Actin	XM_006715764	CCATCATGAAGTGTGACGTTG	ACAGAGTACTTGCGCTCAGGA	173

### Protein quantification

CD24, CD44, peroxisome proliferator-activated receptors type gamma (PPARγ) and actin isoforms were analyzed by Western blot. Protein extracts were obtained from xenograft homogenates using RIPA buffer and mini-Complete EDTA protease inhibitor (Roche Diagnostics GmbH, Germany). Proteins were quantified by the Bradford method (Bio-Rad protein assay; Hercules, CA, USA). Samples containing 50 μg of protein were resolved through 15% SDS-PAGE and transferred to nitrocellulose membranes (Bio-Rad; Hercules, CA, USA) by electroblotting. Membranes were washed with TBS (Tris-buffered saline, pH 7.5) and blocked with 5% non-fat dry milk in TBST (TBS + 0.05% Tween 20) for 1 h at room temperature. Membranes were incubated overnight at 4 °C with anti-CD24, anti-CD44, anti-PPARγ and anti-Actin isoform antibodies (Santa Cruz, Santa Cruz, CA, USA). Antibody binding was detected using horseradish peroxidase secondary antibodies, and the detection was performed using an enhanced chemiluminescence (ECL) system (Bio-Rad; Hercules, CA, USA) according to the manufacturer’s instructions, followed by exposure to radiographic film (Dry Medical X-ray Film, FujiFilm, Edo Mexico, Mexico). Densitometry was performed using ImageJ software (version 1.41, National Institutes of Health, Bethesda, MD, USA).

### Statistical analysis

Data in vitro are representative of three independent experiments by triplicate. The incidence of xenotransplanted mice treated with iodine was analyzed using 2 × 2 contingency tables and a Chi-square as post hoc test. The effects the other analysis were evaluated using Student’s t-test or one-way ANOVA followed by Tukey’s test. Values with *P <* 0.05 were considered statistically significant.

## Results

### I_2_ supplementation disrupts cancer cell viability, decreasing epithelial-mesenchymal transition markers and the invasive potential

**Fig. 1:**
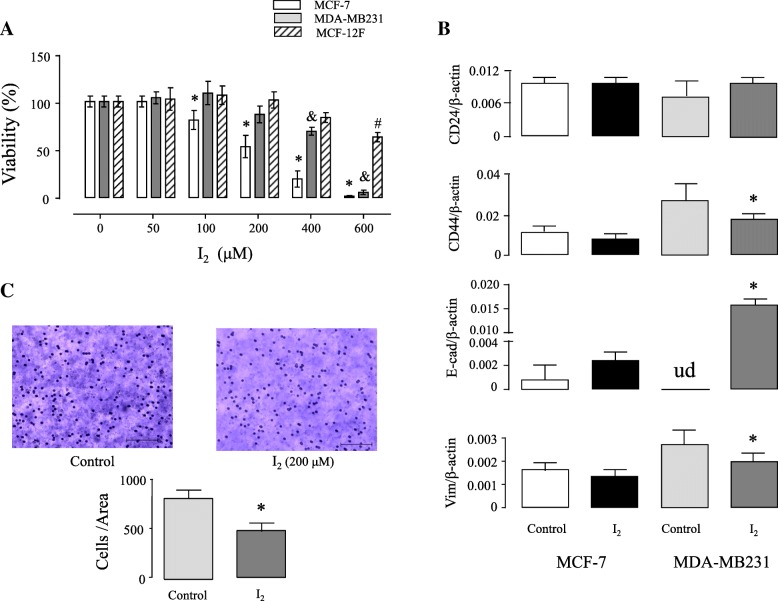
Effect of molecular iodine (I_2_) on viability, epithelial-mesenchymal transition (EMT) and invasive potential in normal (MCF-12F) and cancerous (MCF-7 and MDA-MB231) mammary cells. **a**, Percentage of viable cells after incubation for 48 h in media containing iodine at the indicated concentrations, as determined by the 3-(4, 5-dimethylthiazol-2-yl)-2, 5-diphenyltetrazolium bromide (MTT) assay. Significant differences in * for mammary cancer MCF-7 cells, ^&^ for mammary cancer MDA-MB231 cells and in ^#^ for normal mammary epithelium MCF-12F cells (*P* < 0.05) between their respective control (one-way ANOVA and Tukey’s test). **b**, Cancerous cells incubated for 48 h in media containing 200 (MCF-7) and 400 (MDA-MB231) μM I_2_ were analyzed for mRNA expression for EMT markers CD24, CD44, E-cadherin (E-cad), and vimentin (Vim) by quantitative real-time PCR (RT-qPCR). Values were normalized to the amount of β-actin mRNA amplified. Ud, undetectable; *, significant differences vs. the control (Student’s t-test; *P* < 0.05). **c**, The invasiveness assay was performed to calculate the invasive capability of MDA-MB231 cells in presence of 200 μM I_2_. Images were taken at 20X magnification. Scale bar, 100 μm. Number of cells ± SD that penetrated the membrane. *Significant difference between treated and untreated MDA-MB231 cells (Student’s t-test, *P* < 0.05) (*n* = 3 individual samples)

Fig. 1 summarizes the in vitro effects of the I_2_ supplement on cells from normal and cancerous mammary epithelia. Viability analyses (Fig. 1a) show that MCF-7 are the most sensitive cells, since their proliferation is significantly inhibited (30%) at 100 μM I_2_, reaching IC50 at a concentration of 204.7 μM. Tumor cells with a more aggressive phenotype (MDA-MB231) reached IC50 at a concentration of 409.5 μM I_2_. The viability of MCF-12F epithelial breast cells was affected by exposure to I_2_ from 400 μM (10%) and 600 μM (35%). Further analyses were carried out only in tumorous cells. Gene expression analysis were carried out after 48 h of 200 μM (MCF-7) and 400 μM (MDA-MB231) I_2_ treatment. I_2_ exerted significant changes only in MDA-MB231 cells, with decreases in the expression of CD44 (28%) and vimentin (22%). E-cadherin, which was undetectable in this cell type under control conditions, was significantly induced after 48 h of 400 μM I_2_ treatment (Fig. 1b). Invasive capacity analysis (Transwell assay) showed that 200 μM I_2_ is enough to compromise this capacity in MDA-MB231 cells, as 46% of invasive cells per area were diminished (Fig. 1c).

### Oral I_2_ supplementation impaired cancer implantation and tumor growth, thus preventing weight loss

**Fig. 2:**
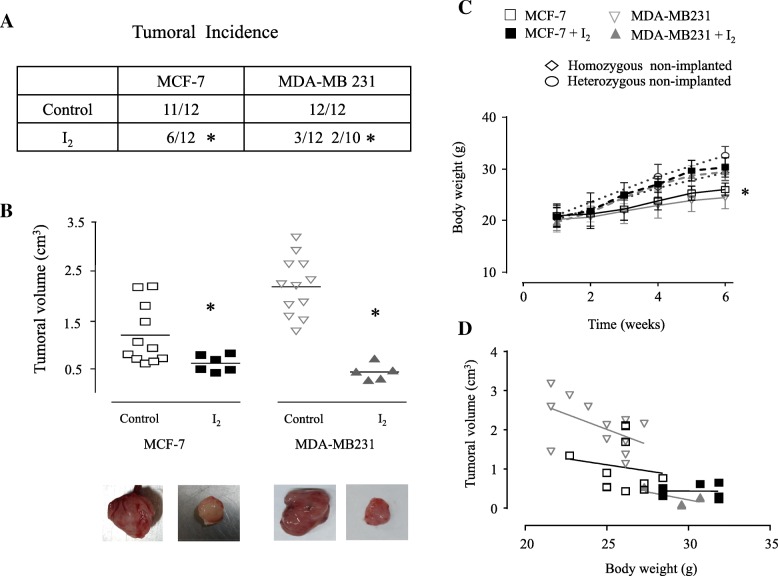
Effect of I_2_ supplement on tumoral incidence, tumoral volume and body weight gain in animals with xenografts of cancer cells. Female athymic homozygous (Foxn1 nu/nu) mice were inoculated with 5 × 10^6^ cells of each cell line in 50 μl PBS and 50 μl Matrigel. The drinking water and the water used for 0.025% I_2_ solution were always deionized. The water supplement (alone or with I_2_) began 48 h after cell inoculation and was maintained throughout the study. Parameters were analyzed after 6 weeks of inoculations. **a**, **Tumoral incidence**. Number of animals that presented observable tumorous mass (0.2 cm^3^). A second group of animals with 10 animals was incorporated to obtain five samples of xenografts from the MDA-MB231+ I_2_ group. * Significant differences between I_2_-treated mice and control mice using Chi-square test. **b**, **Final tumoral volume**. Each dot represents an individual tumor by each group. Data are expressed ± SD. * Significant difference (Student’s t-test; *P* < 0.05) between their respective control. Photographs of representative tumor mass for each group. **c**, **Body weight gain**. Lines represent the body weight gain in animals implanted with xenografts and supplemented or not with 0.025% I_2_ for 6 weeks. The graph also shows the weight recorded for homo and heterozygous non-implanted animals (with and without I_2_ supplement) at 0, 3 and 6 weeks. Data are expressed ± SD. * Significant difference, one-way ANOVA and Tukey’s test. **d, Correlation analysis**. Linear regression between final tumor volume (cm^3^) and final body weight (6th week) in implanted homozygous mice with and without I_2_ supplement. *P* value for MCF-7, 0.6661; MCF-7 + I_2_, 0.9629; MDA-MB231, 0.0852; MDA-MB231 + I_2_, 0.2865. No significant differences were found

For in vivo studies, all animals were manipulated daily for one week before starting the treatments (inoculated with the cell lines [xenografts] and at the beginning of 0.025% I_2_ supplementation in drinking water) to diminish the handing stress. As shown in Fig. 2a, both cell lines in control groups developed efficiently into tumors at an incidence of 91.6% (11 of 12 animals) in MCF-7 and 100% (12 of 12) in MDA-MB231. In I_2_-supplemented animals, the implantation and tumor growth were significantly impaired in both groups: 50% (6 to 12) in MCF-7 and 25% (3 to 12) in MDA-MB231. In fact, in MDA-MB231 + I_2_, a second group of 10 animals (2 to 10) had to be incorporated to obtain at least five samples of tumors from this group (Fig. 2a). Animals continuously supplemented (6 weeks) with 0.025% I_2_ solution developed significantly smaller tumors than those in their respective controls (0.4–0.5 vs. 1.4–2.4 cm^3^), indicating that the presence of I_2_ impaired the tumor growth (Fig. 2b). The animals implanted showed a significant decrease in their body weight gain (18–20%) in comparison with those implanted and supplemented with I_2_ or with the control groups not implanted (homo or heterozygous). The analysis between tumor size and weight loss showed that only the implanted MDA-MB231 group, which developed the largest tumors, exhibited a clear but non-significant inverse correlation (*P* = 0.0852) suggesting the possible establishment of cachexia, known for its association with cancer (Fig. 2d). The necropsy analysis showed that none of the animals exhibited metastasis, adverse conditions in general (ascites or constipation), or visible lesions or metastasis in lung, liver or side-skin of the tumorous region (data not shown).

### The declined expression of EMT, invasive and cachexia markers were related to PPARγ over-expression

**Fig. 3:**
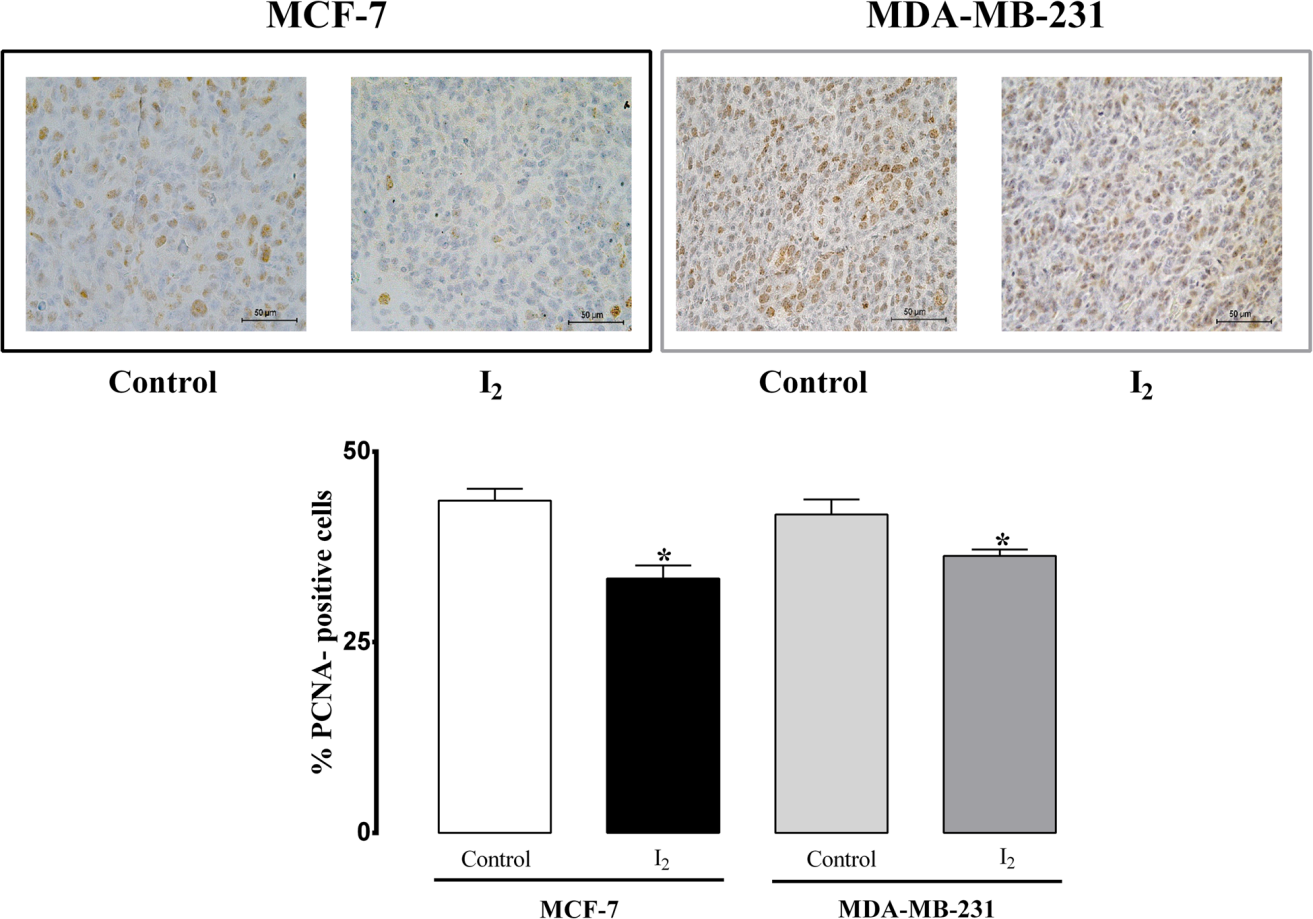
Proliferation rate. Immunohistochemistry showing the presence of PCNA-positive cells in xenografts from animals supplemented with or without 0.025% I_2_ in drinking water for six weeks. Percentage of PCNA-positive cells was quantified by counting the number of labeled cells in at least 500 cells per region, and three random regions were analyzed. Images were taken at 20X magnification. Data are expressed as the mean ± SD (*n* = 6). *Significantly different from its control (Student’s t-test; *P* < 0.05)

**Fig. 4:**
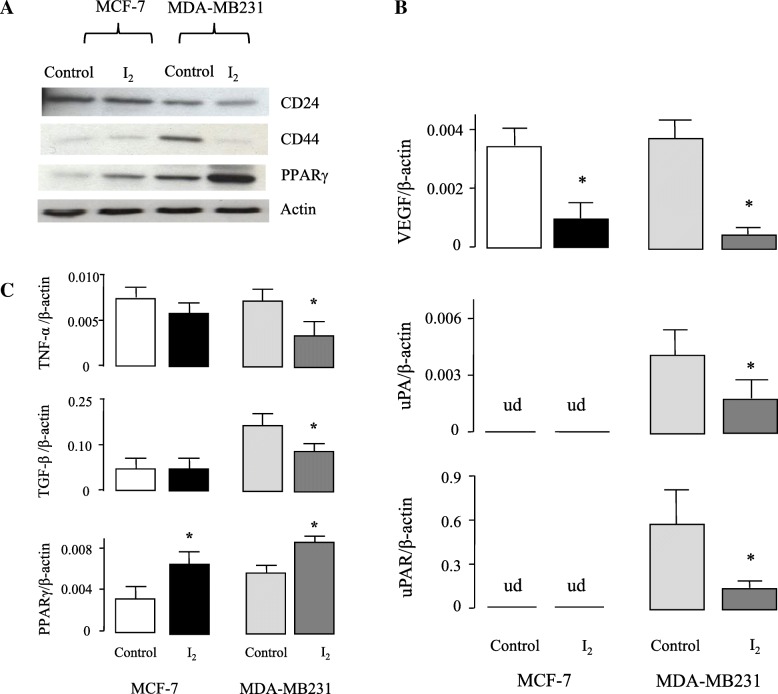
Effect of I_2_ supplement on expression of invasive and inflammatory markers in xenografts. Nude mice were supplemented with or without 0.025%I_2_ in drinking water for six weeks. **a**, Representative Western blot of CD24, CD44 and PPARγ proteins from MCF-7 and MDA-MB231 samples. Equal amounts of protein were loaded on each lane (50 μg), and Actin was run as a control for loading and exposure time (two independent experiments were performed). **b** and **c**, mRNA expression for vascular endothelial growth factor (VEGF), urokinase plasminogen activator (uPA), uPA receptor (uPAR), tumor necrosis factor alpha (TNFα), transforming growth factor beta (TGF-β), and peroxisome proliferator-activated receptor gamma (PPARγ) were analyzed by RT-qPCR. Values were normalized to the amount of β-actin mRNA amplified (*n* = 5–6 individual samples). Ud, undetectable. * Significant differences vs. the control (Student’s t-test; *P* < 0.05)

Xenografts of 0.025% I_2_-supplemented mice showed significant decreases (25 to 30%) in cell proliferation as measured by positive cells to PCNA protein in both cell types (Fig. 3). Also, both I_2_-supplemented groups showed a clear decline in the expression of markers related to stem cell condition (CD24, CD44 protein), invasion (VEGF, urokinase plasminogen activator [uPA], and uPA receptor [uPAR]), immunomodulation (TGF-β), or inflammation/cachexia inductor (tumor necrosis factor alpha; TNF-α), which was more evident in MDA-MB231 + I_2_ (Fig. 4a-c), suggesting that I_2_ actions are related with diminishing the stem condition and invasive potential of these cells. In contrast, PPARγ expression (mRNA and protein) exhibited a significant increase in I_2_-supplemented groups in comparison with control groups, suggesting the participation of these receptors in I_2_ actions (Fig. 4a and b).

### I_2_ supplementation increased the immune antitumor response in homozygous implanted animals

**Fig. 5:**
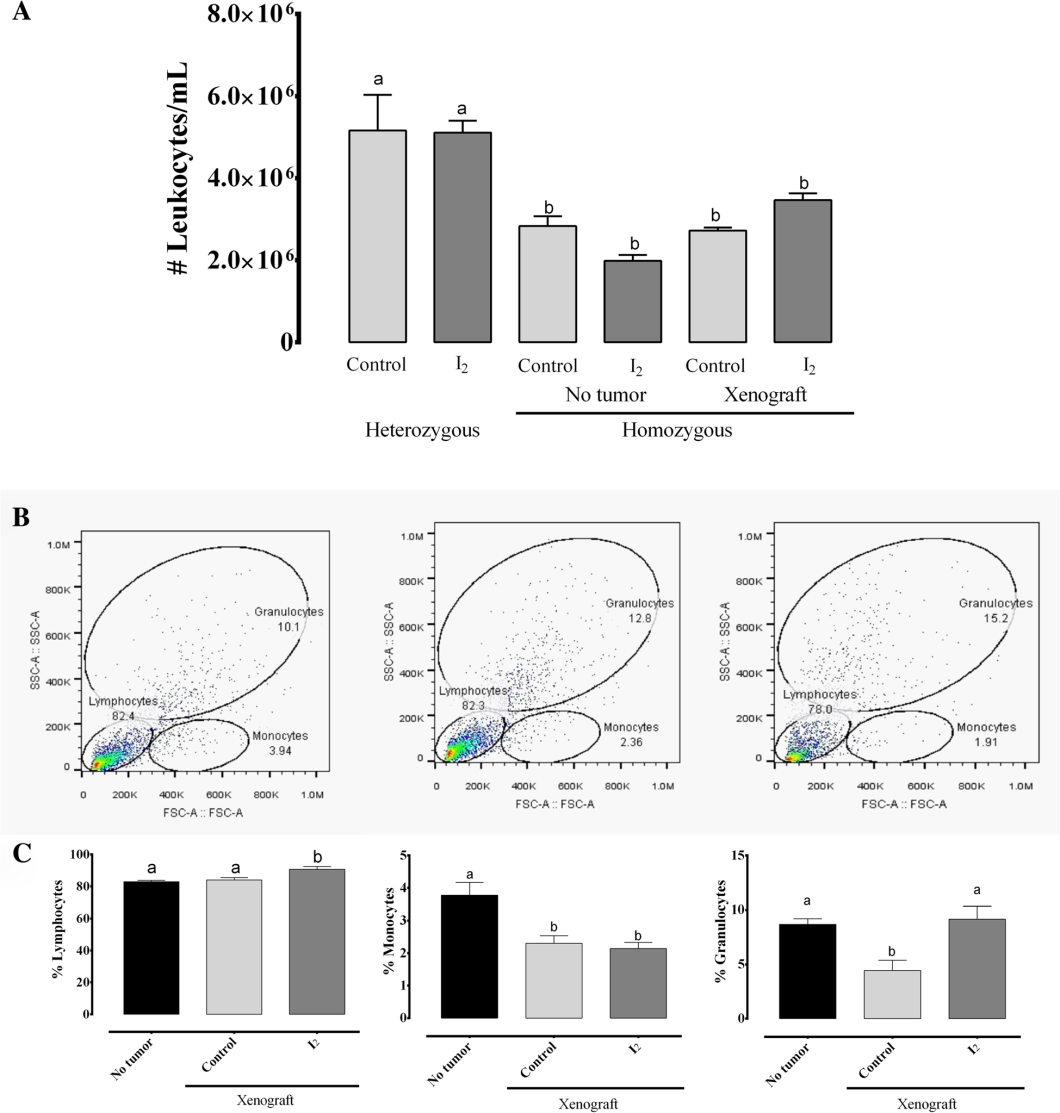
Effect of I_2_ supplement on circulating immune leucocytes. Six-week-old hetero- and homozygous nude mice (with and without MDA-MB231 xenografts) were supplemented with or without 0.025% I_2_ (in drinking water for 6 weeks. **a**, The number of leukocytes in peripheral blood was determined by direct counting after dilution with Turk’s solution. **b**, Peripheral blood populations of leukocytes were separated and quantified by FACS (cytometric images). Differential quantification (lower panel). Data are expressed as the mean ± SD (*n* = 8–10 animals per group). Different letters denote statistical differences (one-way ANOVA, Tukey’s test; *P* < 0.05)

**Fig. 6:**
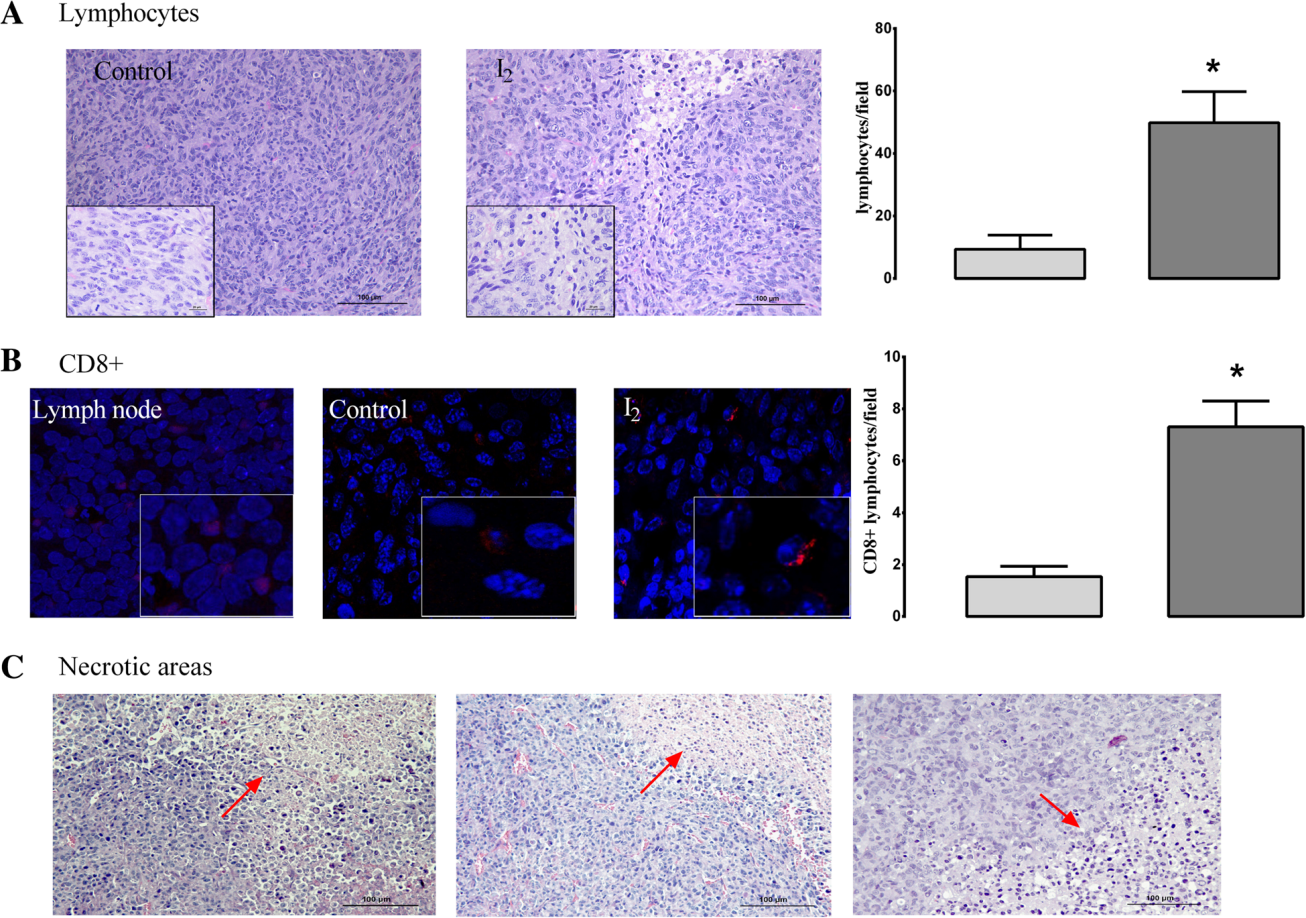
Effect of I_2_ supplement on tumoral immune response. **a**, Micrographs stained with H&E (20X), insert 2X+ zoom of photograph shown lymphocytes (hyperpigmented cells). **b**, Confocal immunofluorescence (63X) of lymph node (positive control) and MDA-MB231 xenografts (Control and I_2_) with antibody against CD8 protein (red). Insert: zoom 2X+ to visualize CD8^+^ lymphocytes (red stain in cytoplasm). Nuclei were stained with DAPI (blue). The analysis was performed as the average of three random fields and the quantification was performed using the ImageJ 1.47 software. Data are expressed as the mean ± SD (*n* = 6). *Significantly different from control (Student’s t-test; *P* < 0.05). **c**, Representative micrographs from necrotic areas (red arrows) from MDA-MD231 + I_2_ xenografts (H&E, 20X)

To analyze the immune response associated with I_2_ supplementation, we compared the circulating and tumor immune cells in control and I_2_-supplemented hetero- and homozygous nude mice. The homozygous animals include groups with and without MDA-MB231 xenografts. As shown in Fig. 5a, homozygous animals exhibited significantly low circulating levels of leukocytes in comparison to heterozygous mice. I_2_ supplementation did not exert any change in heterozygous animals. In homozygous animals, the total leukocyte count had a tendency (not statistically significant) to decrease in non-implanted mice, whereas in implanted ones this concentration tended to increase (Fig. 5a). This last response seems to be related to a rise in the percentage of circulating lymphocytes (12%) and granulocytes (27%) in these groups. Monocytes showed no changes (Fig. 5b). These results agree with the significant amount (three-fold more than control) of lymphocytic infiltration (H&E) observed in tumor samples from I_2_-supplemented animals (Fig. 6a), some of which were positive to the CD8 marker (Fig. 6b). Moreover, the xenografts from I_2_-treated animals showed evident necrotic areas (Fig. 6c). These results suggest the activation of an antitumor immune response secondary to I_2_ supplementation.

## Discussion

In the present report, we analyze the effect of iodine on two types of mammary tumor cell lines that represent epithelial breast cancers. The first is laminal estrogen responsive, which exhibits low invasive potential (MCF-7), and the second is a basal triple negative with elevated invasive potentiality (MDA-MB231). In the in vitro studies, we corroborated previous reports that described that several types of cells exhibited different susceptibility to I_2_, and that the normal types (like MCF-12F) are less sensitive to this halogen [20–22]. This finding has been explained in part by the presence of high concentrations of arachidonic acid in tumorous cells, where I_2_ binds to generate the active iodolipid known as 6-iodolactone, a specific ligand of PPARγ [23]. PPARs are ligand-activated transcription factors initially described as molecular regulators of lipid metabolism. However, PPARγ was recently shown to play a significant role in decreasing cell proliferation and inducing differentiation and apoptosis in many types of cancer [24]. The present results are consistent with previous studies from our laboratory showing that I_2_ supplementation increases PPARγ expression under in vivo and in vitro conditions [19, 25]. The in vitro data showed a marked effect of I_2_ inhibiting the proliferation of MCF-7; however, the anti-invasive gene response (stem and EMT markers) was evidenced primarily in the MDA-MB231 cells, suggesting an important effect of this halogen on the tumorigenic potential. Previous reports have shown that CD44 knockdown in MDA-MB231 cells decreases their metastatic capacity in invasive in vitro assay [26] and that the activation of CD44 results in increased transcription and synthesis of several matrix metalloproteinases, including those in the uPA and uPAR families [27]. The significant decrease in CD44, VEGF, uPA and uPAR expression shown here in tumors from I_2_-treated mice could be additional evidence that PPARγ receptors participate in the antineoplastic effect of I_2_. It has been reported that VEGF and uPA or CD133 and CD44 expressions are inhibited when PPARγ is activated by agonists like rosiglitazone [28] or celecoxib [29], respectively.

The results obtained in vivo show that the significant antineoplastic effect of I_2_ on tumoral growth was accompanied by similar changes in invasive markers observed in vitro, suggesting that tumoral cells maintain their sensitivity to iodine. Interestingly, I_2_ supplementation prevented an 18–20% body weight loss observed in control implanted mice. This weight loss has been described as part of cachexia installation, which is associated with cancer progression, in preclinical [30] and clinical studies [31]. Cachexia is defined as a multifactorial syndrome with alterations in skeletal muscle mass and/or metabolic equilibrium related to an increase in pro-inflammatory cytokines such as TNF-α, interleukin 6 (IL-6), IL-8, and interferon gamma (IFN-γ) [31]. In implanted I_2_-treated animals, the body weight gain was similar to that of heterozygous and non-implanted homozygous nude mice (exposed or not to I_2_ supplement). All these changes in weight were not related to the tumor size or to deleterious conditions (ascites, constipation or metastasis), suggesting that iodine supplementation prevents cachexia to hinder cancer progression by maintaining a small tumor size and decreases markers like TNF-α in tumorous cells.

The present work also analyzes the effect of I_2_ supplementation on the interaction tumor/T cell immune response. The capacity of athymic mice to develop xenografts has been attributed to multiple immunological defects, including reduced interferon-γ production by dendritic cells and functional incompetence of T cells. However, a small proportion of T lymphocyte cells has been detected in these animals, and stimulation with several agents like IL-2 or TNF-α induces the regression of xenografts, indicating the potential reactivation of these cells [32, 33]. Similarly, data in the present work show significantly low circulating leukocytes in homozygous animals, thus corroborating their immunosuppressed condition as well as a clear modulation of the immune system when they were supplemented with iodine. We found that the I_2_ supplement benefits homozygous animals in control conditions (tendency to decrease circulating leukocytes) and in the presence of xenografts to induce activation of the immune system with general (circulating levels) and local anti-tumoral responses (increased CD8+ lymphocytic infiltration and several necrotic areas in their reduced xenografts). The mechanisms involved in I_2_ effects could be explained by the actions of this element. In control conditions is secondary to the rapid and efficient antibacterial effect of I_2_. I_2_ and HIO species are characterized by their high capacity to oxidize biological components like lipids and account for the biocide action of I_2_ solutions [34–36]. In this scenario, circulating I_2_ prevents microorganism proliferation, making an immune response unnecessary or attenuated. In implanted mice, I_2_ could act on tumoral cells through PPARγ activation, forcing re-differentiation or death to reinitiate the targeted immune response [29], or acting directly on immune cells by inducing the reactivation of T cells. In this regard, several reports suggest that the well-known beneficial effect of I_2_ on chronic lesions involves immune responses as well as antibacterial effects [15]. In chronic wounds, its presence activates the influx of macrophages and T cells [37], and under in vitro conditions, I_2_ enhances leukocyte Th1 responses like IL-10, IL8-CXCL8 and IL6 [16]. Moreover, in a recent report from our laboratory we describe that the successful doxorubicin/I_2_ treatment against canine mammary cancer includes the activation of antitumor immune response [38]. The mechanisms by which I_2_ exerts these activations have not been analyzed but could involve the direct antioxidant/oxidant effects or indirect action by PPARγ activation. In the study using metronomic therapy with cyclophosphamide, the antitumor immune response was associated with a consistent activation of the immune cascade (STAT1, interferon and stimulation of cell death), suggesting PPARγ as the main inducer [39].

## Conclusions

I_2_ supplementation decreases the invasive potential of triple negative basal cancer cells MDA-MB231 under in vivo and in vitro conditions. Moreover, oral I_2_ supplementation activates the antitumor immune response in two types of breast cancer cell xenografts (laminal or basal), preventing their tumorigenic progression. Although additional studies with several breast cancer cells type are necessary, the present results allow us to propose I_2_ as a possible adjuvant in breast cancer therapy.

## Additional file

Additional file 1: Description of animal’s conditions and procedures for iodine supplementation. Detailed description of the conditions of the animals used in this study, design of the randomized sample size, protocol for the supplementation of the drinking water with molecular iodine, in addition to detailed description of the quantification of the water consumption. (DOCX 15 kb)
