# Supplementary material for: Molecular iodine exerts antineoplastic effects by diminishing proliferation and invasive potential and activating the immune response in mammary cancer xenografts
Source: BMC Cancer. 2019 Mar 22;19:261. doi: 10.1186/s12885-019-5437-3 (PMC6431076; doi:10.1186/s12885-019-5437-3)
Supplement: Supplementary file 1 — Description of animal’s conditions and procedures for iodine supplementation. Detailed description of the conditions of the animals used in this study, design of the randomized sample size, protocol for the supplementation of the drinking water with molecular iodine, in addition to detailed description of the quantification of the water consumption. (DOCX 15 kb) [file 12885_2019_5437_MOESM1_ESM.docx]

**Description of animal’s conditions and procedures for iodine supplementation**

*Animals*

The animals used in this study came from Harlan Mexico (Mexico City) and were kept in the facilities of our institute that has a specialized vivarium (semi-barrier) to house immunosuppressed animals. The facilities are free of pathogens, and the animal health monitoring program is certified by the SAGARPA (governmental institution of animal health).

*Procedures for determination of sample sizes and randomization:*

Our study contains two groups of animal’s experiments:

Experiment 1. Analyze the effect of iodine supplementation (iodine group) or deionized water (control) in drinking water on the growth of xenografts for 21 days.

Experiment 2. Analyze the effect of iodine supplementation (iodine group) or deionized water (control group) in drinking water for 21 days on the immune response (blood and tumor) in hetero and homozygous mice.

Based on the efficacy of the xenograft (90-100%) and the response of iodine supplementation in previous experiments (with a comparable scheme), we calculated that twelve animals per group are enough to detect at least a modification of 50%, which was the minimum significant difference. In fact, in the group of animals that were xenografted with MDA-MB231 cells, the antineoplastic efficiency of the iodine supplement was so significant (only 3 animals develop tumors) that we required to include another group with ten animals to obtain enough samples for analysis of molecular biology.

Experiment 2. To compare of the immune response between hetero and homozygous animals supplemented with iodine we use as homozygous groups (iodine and placebo supplement) the second group of the MDA-MB231 xenografts from experiment 1, and a group of female’s heterozygotes. In this case, the number used was ten animals per group.

Randomized procedure: Each animal was assigned a random number, thereby generating a list of the animals in entirely random order. The first animal was assigned to treatment group 1; the second animal was assigned to treatment group 2, the third animal was assigned to treatment group 1, etc. until the required number of animals was assigned to each treatment group.

In the follow-up of the experiments, two technicians and/or students weighed and measured tumors in (randomly) half of the animals. One week the placebo group and the other week the iodine group. A third participant received and recorded the measurements of each week.

*Water and molecular iodine consumption:*

We monitor the water/iodine consumption of all animals at the beginning of the first week (Wednesday at 11:00 / Thursday at 11:00 am), and in the fourth week (Wednesday at 11:00 / Thursday at 11:00). We weighed the water bottle on Wednesday and Thursday, the subtraction of grams we divided it among the three animals housed in each box. The average daily consumption of the animals varied between 5.4 ml and 6.7 ml per mouse without significant changes between the groups. The calculation of iodine consumption (0.025% solution) was 1.35-1.62 mg/day/mouse.
